# Supplementary material for: The effect of publishing peer review reports on referee behavior in five scholarly journals
Source: Nat Commun. 2019 Jan 18;10:322. doi: 10.1038/s41467-018-08250-2 (PMC6338763; doi:10.1038/s41467-018-08250-2)
Supplement: Supplementary file 1 — Supplementary Information [file 41467_2018_8250_MOESM1_ESM.pdf]

Supplementary information for: The effect of publishing peer review reports on referee behaviour in five scholarly journals

Giangiacomo Bravo[*], Francisco Grimaldo[†], Emilia López-Iñesta[‡], Bahar Mehmani[§], Flaminio Squazzoni[¶]

Supplementary Methods

Dataset

This document provides additional information on data, methods and analysis, which complements the main text of our manuscript. We have included details on: (1) the protocol for data access and management; (2) the dataset, (3) methods and procedures for gender determination and (4) robustness check of the main findings presented in the manuscript.

Data access and management

Accessing data required an agreement to be established between authors and publishers. A protocol on data sharing entitled "TD1306 COST Action - New frontiers of peer review' PEERE policy on data sharing on peer review" was signed by all partners involved in this research on 1 March 2017, as part of a collaborative project funded by the EU Commission.[1] The protocol established rules and practices for data sharing, which included a specific data management policy, including data minimisation, retention and storage, privacy impact assessment, anonymization, and dissemination. The protocol is available at the following URL: http://www.peere.org/wp-content/uploads/2017/03/PEEREDataSharingProtocol.pdf. Note that while our agreement allowed us to share certain data on the five pilot journals, this was not permitted with the other five journals that were selected from the PEERE database for a robustness check test (see below).

The dataset

The dataset is saved in the enclosed *RevData.csv* file, which includes 62,790 observations and 15 variables. The temporal extension of data was set up according to the starting date and the end of the pilot as follows:
Journal 1: start 2012-01-01, end 2017-03-15
Journal 2: start 2014-11-01, end 2017-12-31

*Department of Social Studies and Centre for Data Intensive Sciences and Applications, Linnaeus University. 35195 Växjö, Sweden.
†Department of Computer Science, University of Valencia, Av. de la Universitat, s/n, 46100 Burjassot, Spain.
‡Department of Didactics of Mathematics, University of Valencia, Av. Tarongers, 4, 46022 Valencia, Spain.
§STM Journals, Elsevier, Radarweg 29, 1043NX Amsterdam, The Netherlands.
¶Department of Social and Political Sciences, University of Milan, via San Faustino 74/b, 25122 Brescia, Italy

Journal 3: start 2014-11-01, end 2016-12-31
Journal 4: start 2014-11-01, end 2016-12-31
Journal 5: start 2014-11-01, end 2017-12-31

The dataset included all the variables listed below. Each missing observation was marked as"NA".

id: unique id for each submitted manuscript; numeric
journal: journal id; factor, 1 = Journal 1, 2 = Journal 2, 3 = Journal 3, 4 = Journal 4, 5 = Journal 5
invitation.date date in which the referee was invited to review; date, POSIXct format
year: the number of years from the first observations; numeric, 0 = 2010, 1 = 2011, ...
open.review: this indicates the open review condition; factor, 1 = No, 2 = Yes
review.complete: this indicates of the review was completed; factor, 1 = No, 2 = Yes
name.published: This indicates if the referee agreed on revealing his/her identity in the published review report; factor, 1 = No, 2 = Yes
recommendation: referee recommendation; factor, 1 = Reject, 2 = Major revisions, 3 = Minor revisions, 4 = Accept
accepted: paper acceptance; ; factor, 1 = No, 2 = Yes
review.time: the number of days from referee invitation to the received review report; numeric $\in [1, 244]$
polarity: polarity of the review report; numeric $\in [-1, 1]$
subjectivity: subjectivity of the review report, numeric $\in [0, 1]$
nchar: length of the review report in characters; numeric $\in [0, 34246]$
reviewer.status: the academic status of the referee; factor, 1 = Professor, 2 = Other, 3 = Dr.
gender: referee gender; factor, 1 = Female, 2 = Male, 3 = Uncertain

Gender determination

Methods for gender determination were inspired by previous research.[2–4] Specific procedures were adapted to the characteristics of our dataset to minimise incompleteness and implicit bias of gender determination techniques.

We first extracted all first names from the database and solved cases of ambiguous or misallocated degrees (e.g., Prof., Dr., Assoc.) and first and middle name initials (e.g., J. Daniel or Daniel J.). We then performed a preliminary gender determination using the following two indicators: (1) the scholar academic title (i.e., Mr., Ms., Miss, etc.), and (2) the output of a gender determination library in Python (gender-guesser 0.4.0 genderize.io), which included 48572 names from 55 countries. The library allowed us to classify each scholars name as male, female, mostly male, mostly female, and unknown.

Robustness check

In order to test our findings, we identified a "control group" of five Elsevier journals in the large dataset collected by the PEERE project which were sufficiently similar to the five pilot journals to ensure the best comparability. Note that the open peer review trial did not originally follow randomisation procedures for journal selection. This means that ensuring a fully consistent experimental procedure for journal comparison was impossible. However, our selection followed coherent criteria aimed to achieve the best inter-journal similarity. The selection criteria were as follows: same discipline/field, similar impact factor, approximate similar number of submissions and submission dynamics (i.e., rate of growth/decline over time) of the five pilot journals (Supp. Fig. 1). As for the pilot journals, in order to avoid learning effect on referee behaviour, we only considered first-round of reviews.

The whole dataset (control + pilot journals) consisted of 138,117 observations, including 21,647 individual submissions. We then included in our model specifications a dummy indicat-

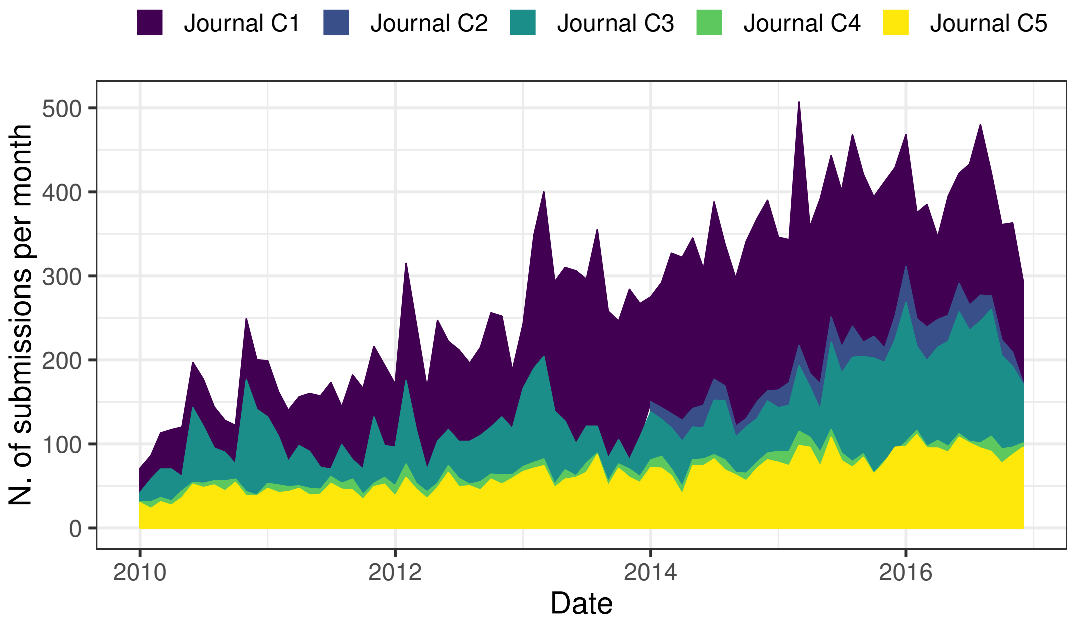

Supplementary Figure 1: Number of submissions per month in the journals used as control group.

ing the sampled control group and estimated three models, which examined the main findings of the manuscript. i.e., the impact of the control group on the willingness to review, referee recommendations, and review time. Note that we excluded from our robustness text the sentiment analysis on referee reports to concentrate on our main findings.

Willingness to review

To check whether referees' willingness to review was different in the two journal groups after the start of the pilot, we estimated a mixed-effect logistic model with referees' acceptance of editorial requests of review as outcome. We included as fixed effects a dummy for the pilot (vs. control) and one for the open review condition, along with the year and the interaction between the pilot and peer review models (open vs. confidential). For the sake of simplicity and readability, we did not include various controls, which were tested in the corresponding model presented in the main text (Tab. 1). We included random effects (intercepts) for submissions and journals. Furthermore, given that the two journal groups (i.e., pilot vs. control) could not be randomised, we explored various dynamics by introducing random slopes at the journal level.

Supplementary Table 1 shows that our findings were robust. Neither the open review dummy nor the interaction term were statistically significant. This suggests that the pilot did not alter the proportion of referees accepting to review. Note that the negative and significant coefficient concerning the year indicated a general decline of referees' willingness to review over time, which occurred also among the pilot journals. The fact that the pilot coefficient was also statistically significant would indicate that the journal control group did not present different dynamics. This confirms that although we could not follow a strict randomisation, the two journal groups were comparable.

Supplementary Table 1: Mixed-effects logistic model on the willingness to review.

Fixed effects	Estimate	Std. Error	z-value	p-value
(Intercept)	0.671	0.211	3.186	0.001
Pilot	-0.735	0.309	-2.379	0.017
Open review	0.123	0.103	1.188	0.235
Year	-0.123	0.006	-20.494	$<$0.001
Open review $\times$ Pilot	-0.150	0.144	-1.040	0.299
Std. Dev. of random effects:				
Submission (intercept)	0.409			
Journal (intercept)	0.510			
Journal (slope)	0.214			
N. of observations	138117.0			
Log likelihood	-88589.8			
AIC	177197.6			

Recommendations

We then estimated a mixed-effects cumulative-link model with similar random and fixed effects by considering referee recommendations as outcome (Supp. Tab. 2). Results indicated that there was no significant difference between the pilot and control group after the start of the pilot. This would confirm that publishing peer review reports did not have any relevant influence on referee recommendations.

Supplementary Table 2: Mixed-effects cumulative-link model on referee recommendations. Note that only observations including completed reviews were considered.

Fixed effects	Estimate	Std. Error	z-value	p-value
Pilot	0.001	0.310	0.004	0.997
Open review	0.014	0.119	0.120	0.905
Year	-0.011	0.009	-1.212	0.225
Open review $\times$ Pilot	0.177	0.168	1.056	0.291
Reject|Major revision	-0.784	0.220	-3.560	$<$0.001
Major revision|Minor revision	0.729	0.220	3.310	0.001
Minor revision|Accept	2.555	0.221	11.577	$<$0.001
Std. Dev. of random effects:				
Submission (intercept)	0.697			
Journal (intercept)	0.480			
Journal (slope)	0.239			
N.	45507.0			
Log likelihood	-58371.3			
AIC	116764.5			

Review time

Finally, we estimated a mixed-effects linear model using review time as outcome. Results of our text confirmed that open peer review review did not influence review time. Note that this was confirmed also examining variable interactions and controlling for the general increase over time of review time. In short, our test confirms that when requested to deliver their report under open peer review, referees did not need extra days compared to the average review time under confidential peer review (Supp. Tab. 3).

Supplementary Table 3: Mixed-effects linear model on review time (days). Note that only observations including completed reviews were considered. Degrees of freedom were computed using Satterthwaite's approximation.

Fixed effects	Estimate	Std. Error	DF	t-value	p-value
(Intercept)	28.907	4.456	8.034	6.487	$<$0.001
Pilot	0.515	6.295	7.995	0.082	0.937
Open review	2.033	1.664	8.808	1.222	0.253
Year	-0.882	0.091	23562.496	-9.749	$<$0.001
Open review $\times$ Pilot	-1.671	2.340	8.557	-0.714	0.494
Std. Dev. of random effects:					
Submission (intercept)	6.326				
Journal (intercept)	9.908				
Journal (slope)	3.521				
Residual	19.058				
N. of observations	44959.0				
Log likelihood	-198527.2				
AIC	397054.4				

References

1. Squazzoni, F., Grimaldo, F. & Marusic, A. Publishing: Journals could share peer-review data. *Nature* **546** (2017).

2. Larivire, V., Ni, C., Gingras, Y., Cronin, B. & Sugimoto, C. R. Global gender disparities in science. *Nature* **504** (2013).

3. Karimi, F., Wagner, C., Lemmerich, F., Jadidi, M. & Strohmaier, M. Inferring gender from names on the web: A comparative evaluation of gender detection methods. In *Proceedings of the 25th International Conference Companion on World Wide Web*, WWW '16 Companion, 53–54 (International World Wide Web Conferences Steering Committee, Republic and Canton of Geneva, Switzerland, 2016). URL `https://doi.org/10.1145/2872518.2889385`.

4. Helmer, M., Schottdorf, M., Neef, A. & Battaglia, D. Research: Gender bias in scholarly peer review. *eLife* **6**, e21718 (2017).
